# Developing an integrated model of care for vulnerable populations living with non-communicable diseases in Lebanon: an online theory of change workshop

**DOI:** 10.1186/s13031-023-00532-x

**Published:** 2023-07-21

**Authors:** Claudia Truppa, Éimhín Ansbro, Ruth Willis, Carla Zmeter, Aya El Khatib, Bayard Roberts, Sigiriya Aebischer Perone, Pablo Perel

**Affiliations:** 1 International Committee of the Red Cross, Beirut, Lebanon; 2 CRIMEDIM - Center for Research and Training in Disaster Medicine, Humanitarian Aid and Global Health, Università del Piemonte Orientale, Novara, Italy; 3 Epidemiology of Noncommunicable Disease Department, Faculty of Epidemiology and Population Health, London School of Hygiene and Tropical Medicine, London, UK; 4 Centre for Global Chronic Conditions, London School of Hygiene and Tropical Medicine, London, UK; 5 Department of Health Services Research and Policy, Faculty of Public Health and Policy, London School of Hygiene and Tropical Medicine, London, UK; 6 International Committee of the Red Cross, Geneva, Switzerland; 7 Geneva University Hospitals, Geneva, Switzerland

**Keywords:** Hypertension, Diabetes, Non-communicable disease, Primary care, Integration, Rehabilitation, Mental health, Multidisciplinary, Patient-centred, Humanitarian, Conflict, Systems thinking

## Abstract

**Introduction:**

The Syrian crisis, followed by a financial crisis, port explosion, and COVID-19, have put enormous strain on Lebanon’s health system. Syrian refugees and the vulnerable host population have a high burden of Non-communicable Diseases (NCD) morbidity and unmet mental health, psychosocial and rehabilitation needs. The International Committee of the Red Cross (ICRC) recently introduced integrated NCD services within its package of primary care in Lebanon, which includes NCD primary health care, rehabilitation, and mental health and psychosocial support services. We aimed to identify relevant outcomes for people living with NCDs from refugee and host communities in northern Lebanon, as well as to define the processes needed to achieve them through an integrated model of care. Given the complexity of the health system in which the interventions are delivered, and the limited practical guidance on integration, we considered systems thinking to be the most appropriate methodological approach.

**Methods:**

A Theory of Change (ToC) workshop and follow-up meetings were held online by the ICRC, the London School of Hygiene and Tropical Medicine and the American University of Beirut in 2021. ToC is a participatory and iterative planning process involving key stakeholders, and seeks to understand a process of change by mapping out intermediate and long-term outcomes along hypothesised causal pathways. Participants included academics, and ICRC regional, coordination, and headquarters staff.

**Results:**

We identified two distinct pathways to integrated NCD primary care: a multidisciplinary service pathway and a patient and family support pathway. These were interdependent and linked via an essential social worker role and a robust information system. We also defined a list of key assumptions and interventions to achieve integration, and developed a list of monitoring indicators.

**Discussion:**

ToC is a useful tool to deconstruct the complexity of integrating NCD services. We highlight that integrated care rests on multidisciplinary and patient-centred approaches, which depend on a well-trained and resourced team, strong leadership, and adequate information systems. This paper provides the first theory-driven road map of implementation pathways, to help support the integration of NCD care for crises-affected populations in Lebanon and globally.

**Supplementary Information:**

The online version contains supplementary material available at 10.1186/s13031-023-00532-x.

## Introduction

The burden of morbidity and mortality due to Non-Communicable Diseases (NCDs) is growing in low- and middle-income countries (LMIC), which are also disproportionately affected by humanitarian crises [1–4]. While evidence on the true NCD burden in crisis settings remains limited, humanitarian actors have gained increasing experience of implementing NCD care (focussing mainly on cardiovascular disease, chronic respiratory disease and diabetes), and the tools and evidence to guide them have grown over recent years [5–11]. However, gaps in high quality evidence on effective care models remain [12]. There is a growing move towards developing more integrated, holistic, patient-centred, high-quality models of chronic care for NCDs in LMIC humanitarian settings, but evidence on the implementation or impact of such approaches (including from the patient perspective) is still limited [7, 10, 13].

The concept of integrated care for NCDs has gained increasing attention globally over the last two decades. Integration may be defined from patient, health system, provider, policy maker or funder perspectives, and may move beyond the health system to include social, education, community and housing services [14–18]. WHO defines health service integration as “The management and delivery of health services such that people receive a continuum of health promotion, disease prevention, diagnosis, treatment, disease-management, rehabilitation and palliative care services, through the different levels and sites of care within the health system, and according to their needs throughout the life course” [19]. Such integration is particularly relevant to chronic conditions, people with multimorbidity, and vulnerable populations, as these often require complex care involving different healthcare disciplines. Integration has been proposed as a means to improve person centredness, and reduce adverse patient outcomes and experience, which may result from the fragmentation of care [14, 15, 20, 21]. Although there are repeated calls for integration of NCD care in humanitarian settings, and a general acknowledgment of its importance, there is a lack of detail on “how to do it” [7–9].

The International Committee of the Red Cross (ICRC) is among the main actors intervening in settings affected by armed conflict and other situations of violence. The organisation’s increasing engagement in NCD care reflects the increasing NCD burden in the countries where it works [3, 22–24]. Its institutional policy and guidance includes four essential principles for the design and implementation of NCD responses: (1) patient-centred care, (2) continuum of care, (3) integrated approach, and (4) sustainability of response through partnership and advocacy [25]. Specific guidelines and tools have been developed to promote the operationalisation of this policy, particularly with regards to integration [26, 27]. However, so far, there has been no formal documentation of their implementation at project level. This study provided an opportunity to document an integrated intervention for NCDs in the humanitarian setting of Lebanon.

### A model of integrated NCDs care in Lebanon: the “CAJA model”

Lebanon has been profoundly impacted by the Syrian refugee crisis since 2011, and is now the country with the highest ratio of refugees per capita in the world [28]. The influx of refugees put additional strain on already weak public essential services. This has been particularly felt in traditionally underserved areas, such as the North East of Lebanon, and it has been compounded over the past 2 years by the cumulative effects of a political and financial crisis, labelled “one of the top three most severe global [financial] crises” globally [29]; a public health crisis triggered by the COVID-19 pandemic; the catastrophic Beirut port explosion in August 2020 [30]; and a resultant health workforce brain drain [31].

Primary care in Lebanon has historically been underdeveloped, and is delivered within a pluralistic, non-standardised system of over 800 primary health centres and dispensaries. Current Ministry of Public Health (MoPH) policy and programmes, supported by international partners, aim to strengthen and standardise primary care provision through the introduction of an accreditation system, shared reporting mechanisms, data collection tools and clinical guidance [32–34].

Since 2014, the ICRC has established a Primary Health Care (PHC) support programme in Lebanon, focusing on areas with the highest concentration of Syrian refugees, which often correspond to Lebanon’s most under-resourced regions with the highest poverty levels and limited provision of essential services. Research from the ICRC and others has previously demonstrated the high burden and unmet NCD prevention and treatment needs among the Syrian and vulnerable Lebanese populations [34–37]. The ICRC PHC programme design evolved to meet this need by including subsidized packages of care for NCDs, targeting diabetes and hypertension, as these conditions pose the highest burden and risk of premature mortality among both Syrian refugees and vulnerable host communities [38]. The programme design was informed by previous evaluations conducted by the ICRC and other humanitarian organisations in Lebanon, and by lessons learned from projects managing HIV and NCD comorbidities in other settings [12, 35, 39, 40]. It was further adapted to the “flat fee” model recently promoted by the MoPH across the whole country, involving a standard, subsidized fee for specific services provided by diverse actors to vulnerable Lebanese and Syrians [41].

**Fig. 1:**
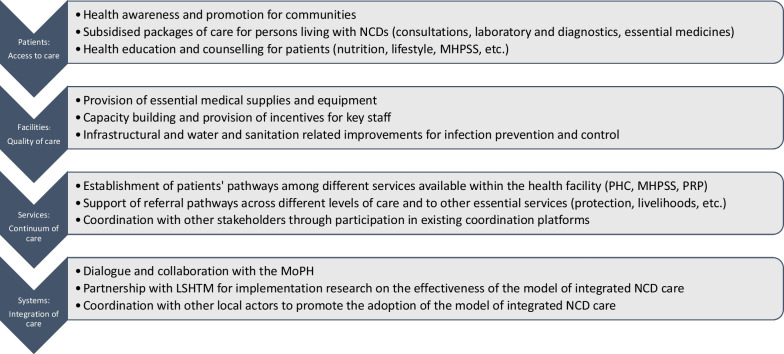
Strategic Framework for integrated NCD care in the ICRC Primary Health Care programme in Lebanon

Since 2020, the ICRC has supported a dispensary run by a Lebanese non-governmental organisation, CAJA (Chabab Al Ataa Al Jazeel Association), in Bireh District, Akkar Governorate. Here, a new operational model of integrated care for NCDs was piloted in 2020, and was named the “CAJA model”. It has since become the standard approach in other ICRC-supported primary care centres across the country. The model operates at four different levels: patients, facilities, services and health system. The activities implemented at each level are described in Fig. 1.

The CAJA model promotes co-location and integration of three different types of services within the same primary care facility: NCD PHC services, Mental Health and Psychosocial Support (MHPSS) services, and Physical Rehabilitation Programme (PRP) services. These three types of services are traditionally implemented as separate projects by different technical teams within the ICRC. In Lebanon, based on the high documented rate of comorbidities, the three teams have designed referral pathways between NCD PHC, MHPSS and PRP services to support the continuum of care internally within the facility and externally with other primary-level or hospital services. Services were co-located in order to multiply the entry points for people living with disabilities, NCDs, and/or mental health conditions into a model of holistic, integrated care, and to better address co-morbidities.

Comorbid mental health and chronic physical conditions are known to lead to poorer physical and mental health outcomes, and, therefore, it is important to identify both conditions and refer for adequate care [42]. MHPSS services are advocated as standard in the approach to NCDs in humanitarian settings but are not yet widely implemented [7, 43, 44].There is also limited experience and no research evidence, to our knowledge, on the integration of physical rehabilitation and NCD care within primary care services in these settings.

In this study, we aim to explore relevant outcomes for people living with NCDs from refugee and host communities in northern Lebanon, along with the processes needed for integration of NCD primary health care, rehabilitation and MHPSS, building on the CAJA pilot project implemented by the ICRC.

## Methods

### The P4C partnership and the opportunity to strengthen the CAJA model of integrated NCD care

To strengthen evidence for the design of models of care for people living with NCDs in humanitarian crises, the ICRC, Danish Red Cross (DRC), and Novo Nordisk formed a partnership entitled Partnering for Change (P4C)—Chronic Care in Humanitarian Crises [7, 12, 45]. The London School of Hygiene and Tropical Medicine (LSHTM) joined as the independent global academic partner. Between September 2020 and February 2021, under the P4C initiative, LSHTM and the American University of Beirut (AUB) conducted a scoping assessment of current ICRC- and DRC-supported NCD care models in Lebanon. The assessment’s findings and discussion with ICRC and partners identified the need to strengthen the existing CAJA model of integrated NCD care. To achieve this goal, in 2021, the ICRC and LSHTM co-designed a follow up research project to identify the necessary pathways to strengthen this model.

### Study setting

The CAJA dispensary (now formally an MoPH-affiliated PHC), is located in Bireh district, Akkar governorate in North East Lebanon. Bireh has an estimated population of 12,000, of whom over 8000 are registered Syrian refugees. Akkar governorate hosts around 12% of the total Syrian refugee population in Lebanon, who make up one-third of the governorate’s population, while half of the host Lebanese population in Akkar Governorate are considered deprived [46, 47]. Akkar performs poorly in most domains routinely monitored in the humanitarian response to the Syrian crisis in Lebanon, with the highest proportion of Syrian refugee households living below the extreme poverty line, poor dietary consumption and diversity, and low rates of birth registration and legal residency status, compared to national averages [48]. In addition, the governorate reports the highest rate in Lebanon of households affected by NCDs (47%), along with the lowest proportion able to access NCD medications [48].

### Rationale for the theory of change approach

A systems thinking approach is increasingly recommended to support operational research in fragile settings affected by complex crises. It is a powerful tool to bridge the gap between theoretical frameworks and the implementation of interventions aimed at strengthening health systems [49, 50]. We identified ToC as the most suitable systems thinking approach for our needs and the research team had previous experience of its use in scaling up interventions in humanitarian settings [51]. ToC is a rigorous and participatory process whereby stakeholders engage in a planning process to articulate their long-term goals and identify a series of linked interventions and underlying conditions that are needed to bring about change [52]. A ToC process maps complex interventions in a non-linear way and can draw out underlying assumptions and highlight hypothetical indirect causal pathways [53, 54]. In LMIC settings, it has been used to design and evaluate PHC strengthening interventions, as well as more specific NCD, MHPSS, and PRP interventions [51, 55–57]. Moreover, it has been adopted to guide the design of integrated models of care, including integrating MHPSS into existing HIV and NCD care programmes [58, 59].

The collaborative, reflective, and iterative nature of the ToC process made it particularly suitable for this study. The ToC process facilitated a research focus on issues deemed important by the team in Lebanon, in a conscious effort to overcome the North–South divide often described in health research in humanitarian contexts [60, 61]. The humanitarian actors had to step out of their comfort zone, and accept balancing the moral imperative to deliver quick operational responses to the affected populations with the additional time and workload needed to document rationales and processes for the chosen approaches [62].

### ToC workshop and map

We conducted the ToC process in 2021 to explore the pathways involved in integration of the three separate strands of care provision (NCD PHC, MHPSS and PRP) within the ICRC CAJA model of integrated NCD Care for Syrian refugees and vulnerable host Lebanese population. It included initial stakeholder meetings with senior ICRC team members based in Lebanon and Geneva, a one day virtual workshop in June 2021 (described below), follow-up online meetings with ICRC staff in Lebanon, and sharing of relevant documents, including ICRC CAJA model project planning and internal evaluation documents. While we had intended that the ToC process would be conducted in person, travel restrictions related to the COVID-19 pandemic forced us to move the process online. To mitigate the drawbacks of a virtual setup, we dedicated additional time for the process, used virtual small groups and break-out rooms, and held repeated follow-up meetings.

The nineteen participants who took part in the one day online ToC workshop included: the ICRC Lebanon Health Team (n = 8), including senior managers and PHC, MHPSS and PRP staff involved in implementation of the CAJA model; ICRC staff from the Health Unit at Geneva headquarters (HQ, n = 5), including specialists in MHPSS and NCDs; and academics with experience in NCD research, mental health, health systems and programme evaluation from Lebanon (AUB), and the United Kingdom (LSHTM) (n = 6). ICRC Health Coordinators/Programme Managers in Libya and Iran with experience in NCD care (n = 2) joined as observers. The workshop was hosted on the Zoom© online platform. It was not recorded to allow for spontaneous exchange and for multiple working groups to meet simultaneously in break out rooms. The LSHTM and ICRC Lebanon teams recorded hand written notes during the workshop, which allowed us to collaboratively develop a summary of the proceedings.

Eight of the workshop participants constituted the core research team: three ICRC Lebanon Health implementing team members (CT, CZ, and AEK), one ICRC headquarters specialist (SAP), and four LSHTM researchers (EA, RW, BR, and PP). Two LSHTM researchers (EA and RW) conducted the workshop and guided the process until the map was finalised. Inclusion of the ICRC implementing team within the research group was in keeping with an embedded approach to co-creation of knowledge using systems thinking [63, 64].

We had intended to involve patient participants, building on their involvement in previous research exploring their experience of NCD care in Lebanon. However, once we transitioned to an online format, patients could not be included since they lacked access to the required information technology.

### Key definitions used during the ToC process

During the ToC process, we developed working definitions of key terms, and refined them in follow-up meetings until consensus was reached within the core research team. Our definition of integration combined patient-centred and health system perspectives: “the coordination, co-location, or simultaneous delivery of quality services (including diagnostic, clinical, rehabilitation, mental health and referral services) to patients living with NCDs, to improve outcomes by overcoming issues of fragmentation, and to allow patients control so that they can access the services they need, or are necessary to achieve outcomes that are important to them, whenever they need them” [65–67]. We defined patient or person-centredness as viewing the health service users as equal partners in planning, developing and monitoring care to ensure it meets their needs. This means putting people and their families at the centre of decisions, considering their desires, values, family situations, social circumstances and lifestyles; seeing the person as an individual, and as an expert, working alongside professionals to achieve the best outcome [20, 68, 69]. We defined a multidisciplinary service approach as care provided by a team of professionals from different disciplines, trained in an inter-professional approach, and engaged in collaboratively providing coordinated care [70–72].

## Results

**Fig. 2:**
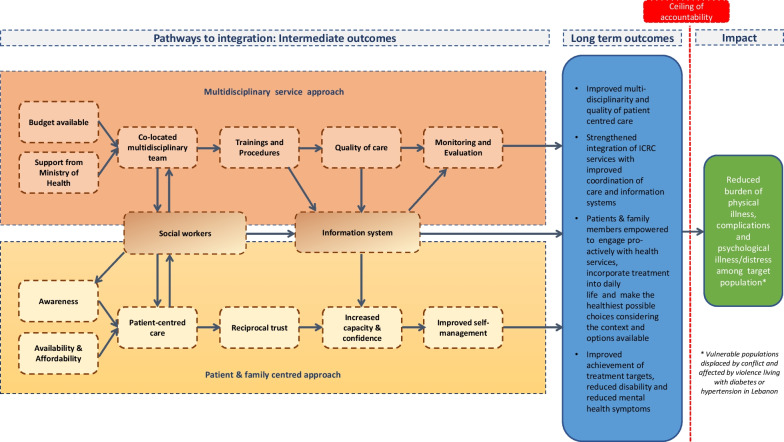
Theory of Change Map representing the pathways to service integration for people living with NCDs attending the ICRC-supported CAJA PHC in Akkar, northern Lebanon (simplified)

ToC participants identified one overall impact for the programme, four long-term outcomes, and two key pathways to achieving these outcomes (Fig. 2), which will be discussed below. We also defined additional elements, including: key interventions, such as the development of a toolkit and standard operating procedures; assumptions, such as the availability of drugs as well as budget for maintaining programme implementation; rationale, rooted in evidence from both the ICRC field experience and experience of other actors in other settings; and indicators for monitoring and evaluation purposes [19, 20, 73]. These are detailed in Additional File 1.

### Impact

Participants first identified the overall impact, that is the real-world change, which they wished to achieve by integrating NCD primary health care, MHPSS and PRP care provision within the CAJA model for the target population. The impact was beyond the ‘ceiling of accountability’ (the level at which the implementing organisation, in this case the ICRC, stop measuring whether outcomes have been achieved as they are beyond the organisation’s ability to achieve that outcome). This is required by the methodology itself, and has been consistently defined as such in similar research [51, 54, 74, 75]. Participants summarised the intended impact as: ‘Reducing the burden of physical illness, complications and psychological illness/distress among the target population (vulnerable populations displaced by conflict and affected by violence and the host population living with diabetes or hypertension in Lebanon)’.

### Long-term outcomes

The long-term outcomes were those which the CAJA care model could achieve on its own (i.e., before reaching the ceiling of accountability). Participants identified four long-term outcomes: (1) improved multi-disciplinarity and quality of patient centred care; (2) strengthened integration of ICRC services with improved coordination of care and information systems; (3) patients and family members empowered to engage pro-actively with health services, incorporate treatment into daily life and make the healthiest possible choices, considering the context and options available; and (4) improved achievement of treatment targets, reduced disability and reduced psycho-social symptoms.

### Pathways

To achieve the expected long-term outcomes, participants identified two main, interconnected pathways: (i) a multidisciplinary (MD) service approach; and (ii) support for the patient and family (Fig. 2).

Following the workshop, the research team finalised the formulation of each element of the ToC map (see Additional File 1) through an iterative, consultative process. The academic partners provided inputs in terms of adherence to best practices, which the implementing partners adapted, considering their operational relevance.

#### Multidisciplinary service approach pathway

The earliest steps identified in this pathway were ICRC headquarters and the country teams’ demonstration of support and provision of adequate budget, in parallel with support from the MoPH and other national level actors. Following from this, the next intermediate outcome identified was the creation of a strong, skilled, co-located multidisciplinary team, including the social worker who would have a clearly defined role and responsibilities to support patients in navigating the system. It was proposed that a new, multidisciplinary way of working would be iteratively co-developed with the implementing team, who would participate in creating tools to deliver MD care, such as operational, training and monitoring tools. These would then enable a well-trained MD team to deliver quality, evidence-based and person-centred care.

#### Support of patient and family pathway

The earliest steps identified in this pathway were to ensure that service availability and affordability for patients were supported, and there were mechanisms in place to increase awareness of these services. This relied on commitment from MoPH and other national actors identified in the multidisciplinary pathway. With these steps established, the next intermediate outcome was for patients and their families to consistently receive empathetic, high-quality healthcare from the whole MD team, which is appropriate to their needs and socio-economic situation. This would enable patients and their families to develop trust in the healthcare team, and to feel respected and legitimised. This trusting relationship provided the basis for patients and their families to strengthen knowledge of their condition and understanding of how the CAJA model services worked. As a result, their capacity and confidence to engage effectively with the health care system would increase and, in parallel, the amount of work required of patients and their families to seek care (or burden of care) would be reduced. The final intermediate outcome of this pathway was that capacity of patients and their families to effectively self-manage their condition, without being overburdened, would increase.

The two pathways converged around two essential elements. The first was the key role of the social worker to support patients and families in navigating the complexities of the Lebanese health care system, to empower them to adopt context-appropriate healthy living changes, and to act as a vital point of contact and facilitator within the broader team. The second was the need for a robust information system that could support the delivery of high-quality, multi-disciplinary, continuous care and facilitate iterative, evidence-based adaptations to the care model.

## Discussion

To our knowledge, this is the first time that a participatory, online ToC planning approach has been applied to services for people living with NCDs in humanitarian settings.

The value of our findings is two-fold. The first contribution is that of expanding the currently limited body of evidence on systems thinking-based methodological approaches to conducting research in humanitarian settings in general, and in the Middle East, in particular [76, 77]. Systems thinking methodologies are increasingly recommended in conducting research in LMICs, as they allow for participatory approaches in the design, implementation, monitoring and evaluation of interventions in complex health systems [49, 78, 79]. ToC has been widely adopted as an implementation research method in LMICs, but to our knowledge, engagement with such approaches in humanitarian settings has been limited. The second contribution is focused on the operationalisation of a new model of NCD care in humanitarian settings. This study proposes implementation pathways to strengthen the integration of primary health care, psychosocial, and rehabilitation services for people living with NCDs, based on ICRC and their partner’s experience in north Lebanon. While there is increasing recognition of the importance of promoting an integrated care approach for people living with chronic disease in crisis settings, there are limited documented examples of how to do this in practice [9, 80].

The ToC process offered several benefits. It allowed for a deeper and shared understanding of key concepts and processes around increasing patient-centred care, through integration of services, a multidisciplinary way of working and greater involvement of the patient and family. It provided a clear map to inform the adaptation of CAJA model activities using an integrated approach. It was also done in a participatory way, involving a range of stakeholders. In addition to the context-specific CAJA programme participants, the ToC benefited from the participation of ICRC headquarters and field staff involved in NCD projects in other countries. The ICRC Lebanon health team’s involvement in the workshop and the related research capacity strengthening activities will ensure sustainability of the approach, allowing better contextualization and faster adaptation of the intervention and research to the emerging needs, especially when the health workforce in the country is under unprecedented strain [31, 81]. The ToC process also triggered a new process within ICRC which will involve greater future participation of patients in programmatic and research design. This is timely given recent evidence that, despite an increased focus on patient-centredness in the design of health policy and programmes globally, the public’s voice is still largely absent in their development [82].

Documenting the ToC process and the resultant map may also support other organisations to operationalise the concept of integration of NCD care in humanitarian settings and the Lebanese MoPH in their current process of integrating MHPSS and NCD care into primary care. This map itself is intended to be a dynamic tool that will evolve with the evidence gained from implementation, monitoring and evaluation of the project. Through the ToC process, we identified two key pathways to achieving greater integration: a support of *patient and family pathway* and a *multidisciplinary service approach pathway.* The *support of patient and family pathway* reflects the growing importance ascribed to person-centredness, a concept that is key to integrated, high-quality health services and is thought to benefit service users, care providers and the health system more broadly [20, 83]. It implies that people requiring health care, their carers and families, should be treated with dignity and respect, and that care should be built around their needs, perspectives and desires, rather than around a specific disease [84]. Person-centred care goes one step further and focusses on the health of communities, empowering them to play an essential role in shaping health services and policies [83].

Within the context of the ICRC’s NCD programmes in Lebanon, the identification of the *patient and family support pathway* was influenced by the organisations’ shift from community-based approaches (whereby humanitarian organisations mobilise and support pre-existing community structures, an approach traditionally implemented in other humanitarian contexts) to adapted, family-centred ones, and by research conducted by the P4C study team, which highlighted the importance of family support for vulnerable patients living with NCDs in Lebanon [80, 85–87]. Limitations of community-based approaches have previously been identified in the Lebanese context. They relate to societal fragmentation; to deeply embedded structural challenges, linked to politically-driven clientelism and predefined power sharing; and to growing intercommunal tensions due to competition over access to privatised essential services and increasingly limited resources [88–90]. The drive to explicitly include patient and family support comes in the context of the highly privatized Lebanese health care system that has historically promoted models of care focused on specialized secondary and tertiary health care, rather than on a holistic, primary-care oriented model centred around patients [91]. The need to strengthen and improve access to person-centred, primary-level NCD care in Lebanon has been widely recognised and is aligned with MoPH and global policy [33].

Workshop participants identified trust in the healthcare team as a crucial factor in this pathway, resonating with research findings from Lebanon and from other countries [88, 92, 93]. The ToC process supported identification of the prerequisites for trust to be developed and maintained, and the mechanisms through which development of trust would lead to increased capacity for self-management, in the wider context of an integrated multidisciplinary system. For example, facilitating referral by holding joint meetings so that the person in whom trust had been developed was present, would enable the existing relationship to be transferred and broadened, rather than the patient and family beginning a new relationship with a different team.

The importance of reducing the burden of healthcare-related activities for patients and their families was highlighted by ICRC project staff, who offered a range of practical suggestions related to strengthening information systems. Specific interventions were proposed to provide for sharing of clinical information if patients moved beyond the CAJA catchment area. Underlying concepts of patient burden and capacity have been explored in frameworks developed to understand how their interactions influence patient behaviour and management of long-term health conditions [94, 95]. The ‘cumulative complexity’ model, which focuses on the balance between ‘patient workload of demands and patient capacity’, supports the pathway identified in the ToC process through which an integrated, multidisciplinary service reduces the ‘work’ required by the patient and family to interact with and navigate the system, and increases their capacity to engage with services, leading to increased capacity for self-management and improved long-term outcomes [95].

Workshop participants considered the second identified pathway, a *multidisciplinary service approach* key to achieving patient-centred, high quality care. Multidisciplinary care has been recognised to be at the heart of integrated care for people living with chronic disease and cancer, especially for older populations affected by multimorbidity. The expertise and skills of different professionals are brought together in a process of interprofessional collaboration to assess, plan and manage care jointly, to provide effective patient centred care along the continuum of care and reduce the burden on individual health care providers [14, 96]. Features of effective MD teams have been previously described, such as the importance of leadership, supporting staff to adopt a multidisciplinary, patient -centred approach through training, which includes communication and relationship building skills, defining and prioritizing relevant person-centred activities, providing practical guidance on embedding the approach into daily practice and, crucially, affording the time and resources to so [20, 97]. An enabling organisational, system and policy environment, including measurement of patient-centred outcomes are also essential [20]. Reflecting these factors, the team proposed incorporating the following elements in the CAJA ToC map: redefining organisational structure and team processes with a clear purpose, assigning roles and responsibilities under a suitable leadership, allocating adequate material and human resources, co-locating MHPSS, PRP and PHC team members in addition to the social worker, coordinating the teams both at CAJA project and central levels in Beirut, setting up clear communication lines, holding team meetings and using common tools for patient identification, assessment, follow-up and referral.

The vital importance of training the multidisciplinary team was emphasised by participants. The key role of interprofessional education has been highlighted in the literature and acknowledged by WHO as essential to preparing health care professionals for collaborative practice. When students from different professions learn about, from, and with each other*,* effective collaboration is enabled, and engagement and partnership with people using health services is fostered, in order to improve patient outcomes [98–100]. Strong hierarchies within teams and “protective routines” professionals may use to reduce the threat to their professional identities can reduce the effectiveness of MD teams [101, 102]. However, mitigation measures may include empowering and showing consideration for all involved through acknowledgement of the importance, specific professional skills and complementarity of each team member’s role. While integrated care provided by multidisciplinary teams is particularly suited to the complex care needs of people with NCDs in humanitarian crises, which can include multimorbidity, psychosocial and protection issues, establishing multidisciplinary care in humanitarian settings in LMICs may be challenging; there may be limited available health care professionals and a lack of experience, guidance or policy to support this approach to care [8, 9, 103].

Improving integration and person-centredness is intertwined with the increasing focus on strengthening the quality of care in LMICs. A Lancet commission in 2018 noted that healthcare quality is low in many LMIC settings and that the most vulnerable, including forcibly displaced persons, are more likely to receive low quality care. Robust, yet simple, data collection which is analysed and acted upon is essential to ensuring quality of care [7, 13, 104, 105]. This is reflected in the emphasis within the ToC map on fit-for-purpose information systems, which link both pathways, supporting coordinated, continuous care, minimising the burden on patients to manage their own information and promoting evidence-based, iterative improvements in the care model.

As other authors have noted, it is essential to consider how a complex intervention interacts with a wider health system and context [53]. It is worth noting that in Lebanon several barriers to implementing patient-centred care have been described: from the fragmentation of the health care system and its heavily hospital-centred approach, to the financial hardship that patients face in a context of worsening economic crisis [29, 41, 106]. The multidisciplinary approach has also been acknowledged to be severely threatened by the cumulative crises in Lebanon that have resulted in a haemorrhage of health professionals leaving the country. Further research will be needed to identify enablers that could enhance health care workforce retention, motivation, and equitable distribution, as well as to document the applicability in Lebanon of strategies such as task sharing, which have proven to be effective in other contexts characterised by high burden of health care demand and low availability of service provision [107]. We intend to document how the ToC is used in practice and to update the map as part of an implementation study of the CAJA integrated model of NCD care.

### Strengths and limitations

The study’s inclusive and iterative, embedded approach ensured both methodological rigour and operational relevance of the findings, which will guide future programme implementation while setting a critical baseline for programme monitoring and evaluation.

Limitations include the fact that the patient and family perspective on integration are missing, due to the online workshop format, in light of the COVID-19 related restrictions and our target population’s limited access to the required digital technology. However, we do not believe our findings are non-representative of patients’ perspectives and priorities. This study took place after, and was informed by, the findings of qualitative research conducted by some of the ToC workshop participants in Lebanon, which identified fragmentation of care and heavy reliance on the family network of support as the main obstacles for continuity of NCD care.

## Conclusion

Applying the ToC process allowed academic and operational teams to explore the characteristics of an integrated model of NCD care proposed for Lebanon, identifying both long term outcomes for patients and their family, and the necessary processes to achieve them. In particular, providing multiple entry-points to care and fostering multi-disciplinary ways of working emerged as critical elements for integration of NCD care services. Despite the virtual process, the teams could explore and come to a common understanding of themes, such as integration, patient-centredness and multi-disciplinary working.

The ToC findings may inform other humanitarian organisations which are increasingly looking at how to develop and strengthen integrated models of care for people living with NCDs in crises. Although there are repeated calls to integrate NCD care in humanitarian settings there is little guidance on how to do it, and this ToC process could provide a useful roadmap to other organisations who are engaging in this discussion.

## Supplementary Information

**Additional file 1:** Detailed theory of change map and legends.

## Data Availability

Data were collected in the form of meeting notes and as iterations of the ToC map. The virtual ToC sessions were not recorded. Materials, such as slide shows and notes, may be accessed by contacting the corresponding author.
