# Supplementary material for: Developing an integrated model of care for vulnerable populations living with non-communicable diseases in Lebanon: an online theory of change workshop
Source: Confl Health. 2023 Jul 21;17:35. doi: 10.1186/s13031-023-00532-x (PMC10360302; doi:10.1186/s13031-023-00532-x)
Supplement: Supplementary file 1 — Additional file 1: Detailed theory of change map and legends. [file 13031_2023_532_MOESM1_ESM.docx]

Supplementary Material

**ToC Map and Legends**

 
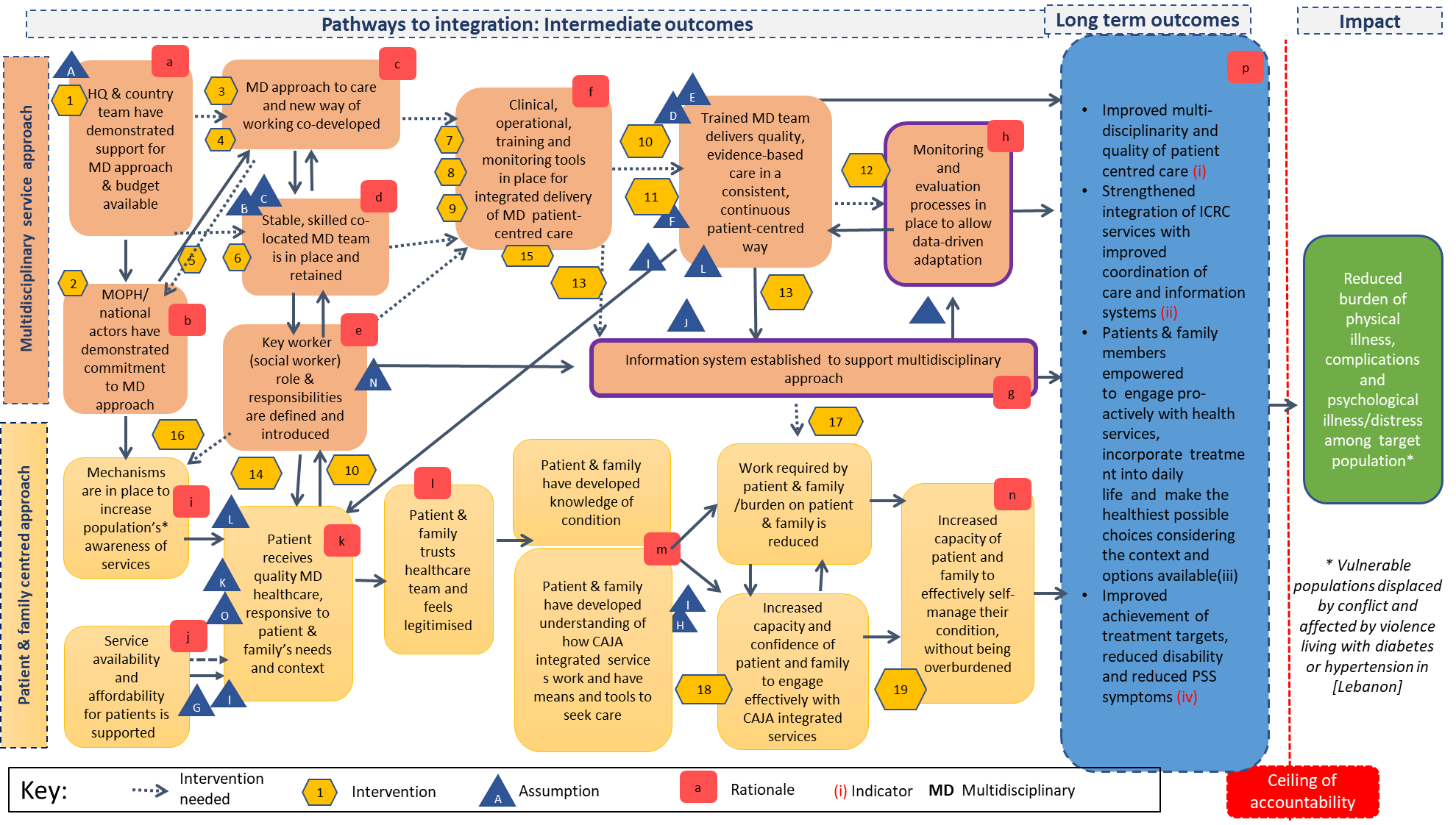


**Interventions**

1. ICRC Internal processes to establish internal support and budget [already in place]
2. Development of concept note to share with facilities/ partners/MOPH
3. Process to define and recruit team, including key worker role
4. Process introduced to collaborate on developing a new, shared approach to multidisciplinary working, to include discussion between ICRC/MoPH & different PHCs, and potential team members at different levels and sites of care delivery
5. Sharing of concept note with MoPH then regular exchanges [via established mechanisms]
6. Strategy/incentive scheme to retain staff
7. Identification of knowledge gaps for staff (both for PHC staff, knowing it might be most effective to target nurses and social workers, promoting task sharing, and for ICRC staff)
8. Development of toolkit for training in multidisciplinary, patient-centred approach to care (evidence-based, incorporating consultation, interpersonal skills training, promoting self-efficacy)
9. Development of guidelines and toolkit for implementation
   1. Starting with sharing of information/tools by different teams and decision on shared package.
   2. Rapid scoping review of existing guidelines to support MDT approach and continuity of care to be performed to identify any gaps
   3. Development of evidence-based SOPs, to define how the existing international/ national guidelines can be integrated in the context.
   4. Creation of new cross-disciplinary MDT tools if needed e.g. shared triage form, referral criteria/pathways, criteria for referral to MDT meeting, SOPs – routine screening of patients with NCDs for MHPSS needs; screening of PRP service users for NCD needs
   5. Clinical/decision support tools supporting early detection,(targeted screening within facilities for defined conditions/patients) secondary prevention, management and detection and management of complications
10. Delivery of start-up training/capacity building; facilitation of access to continuous professional development opportunities (e.g. subscription to journals, participation in congresses etc, integration of new team members
11. Coordination with existing professional orders (LOP, LOPT, LON, etc.) to advocate for granting credits for professionals participating in specific ICRC trainings
12. Robust and meaningful quality improvement process, incorporating patient feedback, is developed and implemented. Process to periodically consider informal and formal feedback from patients, identify gaps and actions to implement changes (important to incorporate this in role descriptions and allocate time)
13. Design, set up, pilot and refinement of information system to support continuity of care and M&E
14. Collaborative planning between patient, +/- family/carers and providers
15. Development of set of joint indicators and integrate into existing M&E tools
16. Build relationship between facility and surrounding community – open days, social media messaging
17. Patient provided with some form of patient held information about their own record, in case they move out of catchment.
18. Patient/family provided with emergency information to take home (emergency contact numbers, red flags) to support self-management
19. Peer groups to support self-care developed and supported (specific peer support groups based on shared needs and criteria, including potentially care givers)

**Assumptions**

1. Staff available with sufficient and appropriate capacity and in sufficient quantity
2. Once established, support and budget will be maintained to sustain programme beyond initial implementation phase.
3. Facility facilitates colocation of services/providers
4. Sufficient time, support, flexibility is allowed for team to learn to work in new ways (both for PHC and ICRC staff)
5. Sufficient, regular time is allocated for MDT meetings and joint assessments
6. Mapping of services exist at national and local levels, by ICRC and working groups under UN umbrella.
7. Services are affordable for patients in target population
8. Patient /family stay in locality and continue to receive care from same facility
9. Key aspects of system remain available and affordable (medicines and equipment) OR social worker/team guides patient/family through changes
10. Information is updated regularly (e.g. as medicine supplies change)
11. Possibility for both patients and family to freely move – {Covid-19 related restrictions, security etc}
12. Sufficient fuel and electricity for staff and patients to reach clinic and clinics to operate
13. A transversal approach between ICRC services is in place e.g. the WatSan team and the Health team work together
14. The key worker has credibility and is “heard” within the broader team
15. Medications are available

**Rationale**

1. Sustained and documented support from ICRC and Lebanon teams is essential for successful implementation at programme level (View of workshop participant)
2. The MOPH, with support of WHO and partners, has undertaken a programme of strengthening and accreditation of Primary Care Centres, and of improving care for physical and mental NCDs at primary level (Hamadeh et al). Commitment from and collaboration with MOPH and partners will help ensure sustainability of this approach, if successful.
3. Multidisciplinary approaches to working are most successful when the shared language, content and processes have been co-developed by the different disciplines involved and specific time has been dedicated to this process (Nolte et al)
4. There are increasing shortages of qualified staff in Lebanon due emigration sparked by the serial crises that have affected Lebanon in the last decade (View of workshop participant)
5. A key worker role has been successful in other contexts, supporting patients to navigate health systems and acting as a key point of contact for patients and an advocate and/or coordinator with broader team members (Nolte et al).
6. Shared tools will help to cement MDT ways of working, while shared training of all involved disciplines will help acknowledge and break down barriers between disciplines, fostering a new sense of shared identity (WHO)
7. Shared information systems, such as a shared paper or electronic patient file, are essential to foster an MDT way of working. Setting up data collection and monitoring systems always takes much more time and energy than anyone anticipates. It's worth investing heavily in data from the outset. It is important to assess existing local systems, train people and have quality assurance in place (Dixon-Wood et al)
8. Quality health care requires regular monitoring and evaluation, with evaluation results feeding into a quality improvement cycle to ensure evidence-based improvements are made (Kruk et al)
9. Populations affected by crisis in Lebanon have low levels of awareness of NCD services, which is exacerbated by the fragmented nature of the country's primary care service delivery (Akik et al, Blanchet et al)
10. Availability and affordability of NCD services in Lebanon is poor, and affordability has worsened given the recent economic crisis and limited donor funding for NCDs (Akik et al, Doocy et al, Willis et al)
11. Patient-centred NCD care is responsive to patients' and families' needs and also adapts to the particular constraints of living in a humanitarian context. (Stewart et al, Boulle et al)
12. Patients' and families' trust in healthcare is an essential component of quality health care (Kruk et al)
13. Patients' knowledge of the condition and understanding of services are essential to facilitate self-care and empowerment, promote trust and foster capacity and confidence to engage with care services (Halepian et al; May et al)
14. There is a balance to be struck between patient empowerment and increasing the burden of treatment on patients and families (May et al). Supporting their engagement with services, facilitating contact with and navigation of primary care and referral services through introduction of the key worker role is key to this (Workshop participants, Nolte et al)

**Indicators**

- An MDT is established with MSW, nursing, medical, physio members recruited and trained by X time
- Clinical consultation (GP), laboratory, counselling/health education (RN/MSW), mental health (psychologist), physio/PRP (physio) services are co-located [or bi-directional referral pathways are in place between sites]ng of services exist at national and local levels, by ICRC and working groups under UN umbrella.
- #/% of people who are satisfied with the quality of support and services received from the ICRC (AAP)
- # total NCD consultations [new and follow up]; new/follow up NCD consultations; # active cohort; exits; loss to follow up;
- #/% of patients with hypertension on BP medication
- #/% active diabetes patients on at least 1 OHG/insulin/OHG +insulin disaggregated by type of diabetes
- MDT global assessment tool is applied to x/% of RPR, MHPSS or NCD patients by the relevant service
- X/% of those internally referred attend the relevant appointment
- X/% of patients internally referred from each ICRC service have had the patient or information returned to site/source of referral
- Means of sharing information about patients between sites and professionals of MDT is established by X time.
- % of missing data for key indicators
- #/% of people who consider that ICRC takes people’s opinion into account when deciding what kind of support to provide (AAP)
- #/% active HTN patients in cohort for 6 months at target BP of <140/90 at last recorded visit
- #/% active diabetes patients in cohort for 6 months at target BP of <140/90 at last recorded visit
- #/% active diabetes patients last recorded HbA1c within last 12 months at target of < 8
- #/% of patients with early detection of neuropathy, vasculopathy or foot ulcer/total foot checks performed
- Significant improvement in psychological distress, mental health symptoms and psychosocial functioning

**References**

1. Hamadeh R S, Kdouh O, Hammoud R, Leresche E, Leaning J. Working short and long: can primary healthcare be protected as a public good in Lebanon today? Conflict and Health (2021) 15:23. Available from: https://doi.org/10.1186/s13031-021-00359-4
2. Nolte E. Implementing person centred approaches. BMJ [Internet]. 2017 Sep 11 [cited 2021 Nov 8];358:j4126. Available from: <https://pubmed.ncbi.nlm.nih.gov/28893736/>
3. World Health Organization (WHO). Integrated Health Services-What And Why? Making health systems work. Geneva.
4. Networks HP, Midwifery N&, Human Resources for Health. Framework for Action on Interprofessional Education & Collaborative Practice. Geneva; 2010.
5. Dixon-Woods M, Mcnicol S, Martin G. Ten challenges in improving quality in healthcare: lessons from the Health Foundation’s programme evaluations and relevant literature. [cited 2021 Nov 8]; Available from: <http://dx.doi.org/10.1136/bmjqs-2011-000760>
6. Kruk ME, Gage AD, Arsenault C, Jordan K, Leslie HH, Roder-DeWan S, et al. High-quality health systems in the Sustainable Development Goals era: time for a revolution. Lancet Glob Heal [Internet]. 2018 Sep 5 [cited 2018 Oct 15];6(11):e1196–252. Available from: <http://www.ncbi.nlm.nih.gov/pubmed/30196093>
7. Akik, C Ghattas, H; Mesmar, S; Rabkin, M; El-Sadr, WM; Fouad F. Host country responses to non-communicable diseases amongst Syrian refugees: A review. Confl Health 13, 8 (2019). Available from: <https://doi.org/10.1186/s13031-019-0192-2>
8. Blanchet K, Fouad FM, Pherali T. Syrian refugees in Lebanon: the search for universal health coverage. Conflict and Health (2016) 10:12. Available from: <https://doi.org/10.1186/s13031-016-0079-4>
9. Doocy S, Lyles E, Hanquart B, the LHAS Study Team, and Woodman M. Prevalence, care-seeking, and health service utilization for non-communicable diseases among Syrian refugees and host communities in Lebanon. Conflict and Health (2016) 10:21. Available from: <https://doi.org/10.1186/s13031-016-0088-3>
10. Willis R, Akik C, Al-Dirani Z, Zmeter C, Truppa C, Aebischer Perone S, et al. Patient experiences of diabetes and hypertension care in humanitarian crises: a qualitative study in Lebanon. BMJ Glob Heal. 2022;Submitted.
11. Stewart M. Towards a global definition of patient centred care. BMJ [Internet]. 2001 Feb 24 [cited 2018 Oct 12];322(7284):444–5. Available from: <http://www.ncbi.nlm.nih.gov/pubmed/11222407>
12. Boulle P, Kehlenbrink S, Smith J, Beran D, Jobanputra K. Challenges associated with providing diabetes care in humanitarian settings. Lancet Diabetes Endocrinol [Internet]. 2019 Mar 13 [cited 2019 Apr 23];0(0). Available from: <http://www.ncbi.nlm.nih.gov/pubmed/30878269>
13. Halepian L, Saleh MB, Hallit S, Khabbaz LR. Adherence to Insulin, Emotional Distress, and Trust in Physician Among Patients with Diabetes: A Cross-Sectional Study. Diabetes Ther. 2018 Apr 1;9(2):713–26.
14. May CR, Eton DT, Boehmer K, Gallacher K, Hunt K, MacDonald S, et al. Rethinking the patient: Using Burden of Treatment Theory to understand the changing dynamics of illness. BMC Health Serv Res. 2014 Jun;14(1):1–11.
